# Hospital and Institutional News

**Published:** 1915-02-27

**Authors:** 


### HOSPITAL AND INSTITUTIONAL NEWS.

### HONOURS FOR ARMY SURGEONS.

The King has been pleased to approve, among others, of the undermentioned rewards for services rendered in connection with operations in the field, dated February 18: Staff: *To be Surgeon-General.*—Colonel (temporary Surgeon-General) R. Porter, M.B. *To be Brevet Lieut.-Colonel.*—H. Ensor, D.S.O., M.B. (temporary Lieut.-Colonel). *To be Knight Commander of the Order of the Bath.*—Surgeon-General Sir A. T. Sloggett, C.B., C.M.G., K.H.S. *To be Companions of the Order of the Bath.*—Surgeon-General T. P. Woodhouse, Colonel M. W. O'Keeffe, M.D., Colonel (temporary Brigadier-General) H. D. E. Parsons, C.M.G., Colonel E. H. L. Lynden-Bell, M.B., Colonel S. Hickson, M.B., K.H.S. *To be Companions of the Order of St. Michael and St. George.*—Colonel R. H. S. Sawyer, M.B., F.R.C.S.I., Colonel F. H. Treherne, F.R.C.S. The awards to the Medical Services are given on page 492.

### THE DOUBLE V.C.: UNPRECEDENTED HONOUR FOR A SURGEON.

In the Supplement to the *London Gazette* the names of the latest recipients of the Victoria Cross are given. There are eleven names in the list, divided in almost equal numbers between the commissioned and the non-commissioned ranks. But among them, placed last in order but inevitably first in honour, is that of a lieutenant in the Royal Army Medical Corps, who has received the unique distinction of the double V.C. To be more precise, a clasp to a Victoria Cross already gained has also been awarded, which is an unprecedented distinction, and one which, one may feel, falls where honour is due—to the Royal Army Medical Corps. The official announcement is as follows: "Clasp to Victoria Cross.—Lieut. Arthur Martin Leake, R.A.M.C., who was awarded the Victoria Cross on 13th May, 1902, is granted a Clasp for conspicuous bravery in the present campaign: 'For most conspicuous bravery and devotion to duty throughout the campaign, especially during the period 29th October to 8th November, 1914, near Zonnebeke, in rescuing, whilst exposed to constant fire, a large number of the wounded who were lying close to the enemy's trenches.' The following is the description of the act of valour for which Lieutenant Leake received the Victoria Cross in 1902: 'While tending the wounded under heavy fire from forty Boers at 100 yards' range at Vlakfontein he was shot three times: but although grievously hurt he refused water to relieve his own thirst until all the others (eight) had had some.'"

### THE RETENTION OF POST-MORTEM SPECIMENS: THE LEEDS INFIRMARY CASE.

A matter of some interest to most medical men and to all hospital staffs was mentioned in the course of a compensation case at Barnsley County Court. The man whose death led to the claim died in the Leeds Infirmary, and a *post-mortem* examination was made. In the medical evidence it was stated that the heart was kept for scientific purposes. In commenting upon this, Judge Fossett Lock said that it was no doubt important for teaching purposes that specimens should be preserved, but that was no reason for stealing. Scientists were not entitled to break the laws of their country and to ignore the principles of morality. He was bound to say that the Leeds Infirmary had broken the law in this case. The Judge's comments will come as a surprise to medical men. The right to retain internal organs at a *post-mortem* for scientific purposes has been generally assumed. It does not seem reasonable, indeed, to adopt any other attitude.

### THE GOVERNMENT AND INFIRMARY COAL SUPPLIES.

The Metropolitan Boards of Guardians are experiencing, along with all other public authorities, great difficulty in obtaining coal supplies. At Southwark, for instance, the contractor could send none in, with the result that at the infirmary the coal supply was reduced to eighteen tons, whereas fifteen tons are consumed in a week. At Lewisham experiments have been made which show that in the day-time coke can be burnt, thus reducing the consumption of coal, but, owing to the difficulties of cartage, even coke is difficult to get. At Camberwell arrangements are being made for the storage of from three hundred to four hundred tons. The Metropolitan Asylums Board have induced the Admiralty to come to their aid in obtaining a supply of coal, and, if the Admiralty are prepared to help one authority, they should be induced to come to the aid of all the hospitals and infirmaries. A suggestion is to be made to the Local Government Board that they shall provide a central store for London, from which the stocks can be drawn, and, in view of the difficulties there are in getting other things, especially flour and provisions, the store might be extended to include those goods as well. There is little doubt that public authorities are being badly exploited, as well as the people, by contractors. As soon as the latter are given an undertaking that they will be paid enhanced prices, they find coal where none existed before; whilst when they are promised extra for cartage, coal is delivered in a few hours. These have been the experiences of the Southwark and Camberwell Boards of Guardians. The Government control of supplies for Metropolitan institutions would do away with many of the temptations to extortion now open to contractors.

### THE DEATH OF A GREAT MATRON.

We regret to record the death of Miss K. H. Monk, formerly the beloved and respected matron of King's College Hospital, from which post she entered into a well-earned retirement in April 1906. The best public illustration of regard for her powers of administration, and respect for her character and achievement, took place quietly a few months later, when Lord Methuen, as chairman of the committee of management of King's College Hospital, headed a deputation which proceeded to Miss Monk's residence at Brighton in order to present her with a testimonial, in the form of a purse of money, subscribed by five hundred personal friends and representatives of the committee and staff of the institution. We need only here recall that Miss Monk held her responsible office for nearly twenty-three years, a period, it will be recalled, filled with generous activity and progressive work in the rapidly growing scope and branches of her profession. Perhaps the best brief summary of her activities can be given in the few words which Miss Monk published in the *Nursing Mirror* (then a section of The Hospital) in acknowledgment of the above honour. Writing in the third person Miss Monk remarked that it was "a subject of deepest regret and sorrow that her failing health should have prevented her finishing the work that she had hoped to accomplish for the great cause of our hospitals and the care of our sick. She especially regrets that she should have been unable to continue her services to the committee at this critical period in the history of King's College Hospital, and to see the completion of the removal of the hospital to Camberwell before her retirement. It is also a great sorrow to her to be compelled to sever her connection with Queen Alexandra's Imperial Military Nursing Service, more especially as Her Majesty had recently paid her the further compliment of placing her on the Council of the British Red Cross Society, in both of which works she took the warmest interest." Much more might be written of her work for nurses and the nursing profession, but hospital men will remember her as a fine spirit and a rare administrator, who lived and practised those ideals of nursing and personal character of which Florence Nightingale will always remain the type. A personal memoir of Miss Monk appears on page 493.

### FLORENCE NIGHTINGALE'S STATUE UNVEILED.

On Wednesday morning the statue of Florence Nightingale was informally unveiled, and we now see the result of the labours of the Memorial Committee and the completion of half of their scheme. Viscount Knutsford first suggested to the committee the effect that might be produced by associating with the Crimean Memorial this new statue and the statue of Sidney Herbert, the War Minister responsible for sending Miss Nightingale to the Crimea. This question of site had been one which had given the committee much concern. The Office of Works adopted this suggestion, and the committee owe much to the First Commissioner, as well as to Mr. Lionel Earle, C.B., for the great assistance which was given by the Office in carrying out the committee's intentions. We now have a unique group in Waterloo Place which has undoubtedly added to the beauty of one of our prominent sites, the alterations to which we have already described in previous issues. The statue of Florence Nightingale is the work of Mr. Arthur G. Walker, and presents a very noble figure. It was on Friday, March 31, 1911, that the Lord Mayor presided over a great meeting at the Mansion House when the establishment of a Memorial to Miss Nightingale was agreed to on the proposition of Viscount Haldane, at that time Secretary of State for War. The committee appointed to carry out the objects of the Memorial had two objects in view: To erect a statue in a suitable place in the Metropolis; and to provide annuities, to be known as the Florence Nightingale Annuities, for trained nurses who while devoting the best years of their lives to following their vocation have been unable to provide adequately for their old age or infirmity. As the son of the War Minister who sent Miss Nightingale to the Crimea no more appropriate selection could have been made than that of the Earl of Pembroke as chairman of the Executive Committee. He put his heart into the matter, and with the assistance of Mr. G. Q. Roberts as honorary secretary a wide and successful appeal was made for help from nurses, sailors, and soldiers throughout the world. As to the beautiful statue itself, the Early Victorian gown would not be accepted in the present day as a nurse's uniform, but the artist's choice is none the less justified. It has been incorrectly stated that the statue of Florence Nightingale is the first statue of any woman, other than that of a queen, to be erected in London. But she was a queen among nurses, and as such we honour her. No more appropriate time than the present could have been found for the unveiling, but it was felt that a public unveiling could only be worthy of Miss Nightingale if it were a great ceremony. As the present is no time for that, need we regret that the memorial statue should be uncovered without formality? The statue is only the symbol of her finest memorial —the work now going on daily for the wounded in so many hospital wards.

### THE NIGHTINGALE ANNUITIES SCHEME.

The second part of the Memorial is one which must enlist the sympathies of many. It is pleasing to be able to record the fact that no less than £4,000 has been handed over to the committee of the Trained Nurses' Annuity Fund, of 73 Cheapside, to form the annuities which were aimed at in the original appeal made, and it is a satisfaction to know that already six annuitants are benefiting under this fund.

### THE FIRST LONDON COUNTY COUNCILLOR TO BE WOUNDED.

The first member of the London County Council to be wounded during the present war is Lieut. Courtauld, who is one of the representatives of North Lambeth. Lieut. Courtauld is a past chairman of the Midwives Act Committee and has been closely identified with the work of the Public Health Committee. At the outbreak of hostilities Mr. Courtauld volunteered for service with the Royal Army Medical Corps.

### CASUALTIES IN THE GERMAN MEDICAL SERVICE.

Up to the first week in January the casualties in the German Medical Service had been heavy; 132 medical men had been killed, 45 had died, and 166 were missing or prisoners. Among the killed were at least three distinguished men to whom the Berlin correspondent of the official organ of the American Medical Association refers. One of the eminent men referred to is Professor Jochmann, the medical head of the infectious diseases department of the municipal Rudolf Virchow Hospital; he succumbed to typhus fever which he is said to have contracted while treating Russian prisoners. He was only forty years old, and the author of a text-book on infectious diseases which was published a few weeks ago, and is said to be the leading work of its kind in German literature. Professor Sprengel, the superintendent of the surgical department of the Ducal Hospital in Brunswick, died from sepsis, as a result, it is said, of operating on a wounded soldier. Professor Jakobi died in the field as the result of disease. He produced an Atlas of Skin and Venereal Diseases and was one of the editors of "Iconographia Dermatologica." Iron crosses have been generously distributed among physicians and surgeons, nearly three thousand of whom have been decorated.

### A STAFF SCARCITY AT WORCESTER INFIRMARY.

Having advertised without success for a resident medical officer, Worcester Infirmary committee agreed to advertise for either a male or female doctor. Subject to the approval of the committee, Dr. Martha Stewart, of Belfast University, has been already appointed. She is now in residence, and the chairman of the committee has reported that her work is very satisfactory. Her appointment has been approved. Mr. Park read a statement of the financial position at the recent monthly meeting, which gave an analysis of the income from subscriptions. The result showed that the total receipts from subscriptions amounted to £1,704 in 1914, compared with £1,469 in 1913, a valuable increase of nearly £300. Mr. Park thought that some formal recognition should be made of the services Mr. Bates, sen., had rendered to the infirmary during the time they were unable to obtain a resident medical officer. A hearty vote of thanks to him was carried unanimously.

### THE BIRMINGHAM SYSTEM OF VOLUNTARY AID.

It is now thirty-four years since Birmingham was constituted a local centre of the St. John Ambulance Association, and the growth of the work has been most remarkable. In round figures 10,000 men have completed a course of instruction and very nearly an equal number have received certificates, while the numbers of women instructed and certificated are over 2,000 and over 1,000 respectively. The work has grown on all sides, and it is significant of its reality that "decent headquarters" are still asked for by those acquainted with its activity. The Association, in fact, now has a brigade of 700 workers who are qualified to give continued besides first-aid care to the wounded. Classes, examinations, certificates, are supplemented by the different work of the various sections, one of which is now responsible for the rest station at Birmingham, by means of which the 20,000 wounded who have passed through the city have received comforts and refreshments at the different railway centres. The Association has carried on at private cost a hospital at Richmond Hill, with accommodation for thirty patients, and two other hospitals are being mobilised at Lordswood House and Erdington. Nearly a hundred of the men have joined the Colours, and an equal number have volunteered. It is also significant, as the Lord Mayor, Alderman Bowater, recently pointed out, that no money has been allocated from the London funds to the branch at Birmingham. There are now seven Voluntary Aid Detachments in Birmingham, and the hospitals for which they are responsible, alluded to above, formed useful appendages to the 1st Southern General Hospital, and have received the sanction of the General Officer Commanding.

### DR. E. C. AUSTIN'S NEW POST.

All interested in the progressive standard of Poor-Law infirmary work will have noted with interest the appointment of Dr. E. C. Austin, medical superintendent of the Hope Hospital of the Salford Union, to that of medical officer of the Willington Institution at a salary of £700, rising to £1,000 per annum. Readers who recall events described in former issues of The Hospital will remember that the Hope Hospital previous to Dr. Austin's appointment was an example in certain respects of a state of things not conformable to the modern standard of infirmary administration, owing to a reactionary element among the Guardians which did not appreciate the urgency of reform. Dr. Austin, who succeeded a capable but transient predecessor, had a difficult task before him, and the measure of success which he achieved shows how much depends upon the medical superintendent in maintaining the standard and resisting reaction.

### NORTH EVINGTON INFIRMARY AS A SUPPLEMENTAL BASE HOSPITAL?

The suggestion to make Leicester a hospital centre for the accommodation of 2,000 additional sick and wounded soldiers, which, as was intimated in our columns last week, would bring the hospital accommodation of Leicestershire to about 3,000 beds, has been rapidly developed during the week by a visit from General Kenny, the Deputy Director of Medical Services of the Northern Command. In a course of inspection (the De Montford Hall and six of the principal Council schools were selected as suitable for hospital purposes) it was suggested that until further communication, the buildings should be continued to be used for their present purposes. Moreover, the Local Government Board have suggested to the Leicester Board of Guardians that the North Evington Infirmary may be required for military purposes, and have asked the local Board's consent to this course. The letter is being considered by the Board this week. The alternative to the acquisition of decentralised buildings now filling important functions was the enlargement of the 5th Northern General Hospital, which, standing on a site of 33 acres, appeared to possess ample room for extensive additional buildings, an alternative, be it said, which found strong favour locally. Doubtless, however, the military authorities have reasons, although these are not very apparent, for the selection of buildings which must obviously be structurally inadequate for hospital purposes.

### ADVANTAGES OF SUCH A SCHEME.

The acquisition of the North Evington Infirmary has, on the other hand, much to commend it, for qualified observers point out that it would be quite a workable plan to make this institution a supplemental base hospital—the fine situation of the institution and the large acreage available for any extensions are all insisted on as making it a very desirable building to acquire. Presumably the War Office authorities have already considered the necessary means for securing adequate and efficient medical and nursing services, as to which, as our correspondent pointed out, there are many inherent difficulties. So many important considerations are, in fact, involved that these plans cannot but be awaited with great interest.

### THE REARRANGEMENT OF LOCAL ADMINISTRATION.

The measures to be adopted by the local authorities in the extensive and almost complete rearrangement of the town services of Leicester will be of more than local interest in view of the fact that inquiries are being made in other large centres for further hospital accommodation. The selected buildings not being immediately required, the educational and town authorities will be able to formulate their plans to meet the exigencies involved, of which they will doubtless give early intimation. Especially will the proposals of the Leicester Board of Guardians be awaited with interest, involving, as they probably will do in the light of the proposals mentioned in the previous paragraph, the arrangements for the accommodation of the 700 inmates at present under their care. We suggest, however, that with all the space available at the base hospital and the North Evington Infirmary it is undesirable, save in emergency, to allow school buildings to be used for hospital purposes. An early conference should be held representing all the interests involved. Leicester, which has given the lead so often, will be eagerly looked to by other provincial centres for a solution to the problem it is now facing and other towns are about to meet.

### MENTAL HOSPITAL LAUNDRY WORK.

Some indication of the growth of the work at Colney Hatch Mental Hospital is afforded by a report of the London Mental Hospitals Committee explaining its decision to instal an additional washing machine at a cost of £160. There are 126 more patients resident there now than there were in 1909, and 390 more than in 1904, and a further increase is expected in the immediate future. This has occasioned an increase in the number of articles which have to be dealt with in the laundry. The bed-linen and underclothing are now changed much more frequently than they used to be, as more patients are kept in bed for observation, more careful methods of disinfection are in use, and generally the nursing and sanitary conditions have been greatly improved. The custom of washing certain articles in the wards, which once obtained, has been discontinued, and for all these and various other reasons the work which the laundry is required to do has greatly increased, the approximate weekly average of articles dealt with being 57,000, as against 47,000 in 1911. The present machines are not speedy enough to turn out the necessary work in the proper time, and as a result great congestion takes place.

### THE EDUCATION OF THE HOSPITAL OFFICER.

We publish elsewhere in this issue an interesting letter from Mr. G. Watts, secretary of the City of London Hospital for Diseases of the Chest, in which he discusses some of the questions raised in our recent leading article on the subject of "Hospital Experience and the Higher Appointments." He makes this article a text for a discussion on the present facilities for hospital training, and criticises our remarks on those professional organisations which admit everyone who can claim, in however small a degree, to be eligible for membership. Mr. Watts admits the difficulty consequent upon a proper desire to be representative, but argues that we overstate its bad effect in existing circumstances. As he correctly concludes, the degree of impolicy obtaining must be a matter of opinion, and it is not on the precise line which ought to be drawn that we want now to insist. Larger questions are at stake, which may be summed up in the present admitted need for improving the training of junior hospital officials. The Incorporated Association of Hospital Officers, Mr. Watts reminds us, is taking the matter in hand, and a statement of the conclusions arrived at by that body is shortly to be expected.

### THE PRESENT DISCONTENT.

Certainly the time is ripe for a discussion, since it would be idle to deny that there is a considerable degree of heartsearching in the rank and file of the profession about the prospects offered by hospital work at the present time. At the senior end of the scale the long-discussed question of a recognised scheme of pensions, in which men like Mr. Michelli have long been interested, is pressing for solution. Among the junior men the prospects, chances of promotion, and rewards for experience have lately been very much canvassed. It was, we believe, the general question rather than the particular disappointment which caused the controversy, which we have ventilated, and other less open expressions of opinion, over the recent

Hampstead and Birmingham appointments. Communications still reach us over the former, but mainly, as we regret to see, from correspondents who desire to remain anonymous. We have published several, but the fact that many would-be writers are anxious to hide their light under a bushel while expressing strong views argues surely a lack of responsibility, which, even allowing for the nature of the controversy, is an unfortunate sign for the profession. That is why we welcome Mr. Watts' letter, and hope that his points may be taken up as frankly as he has stated them, so that grievances and criticisms of the existing system of training, and helpful views for improving it, may be debated in our columns.

### FIFTY YEARS AS SECRETARY.

To outstanding institutional records of long service must be added the period of fifty years now concluded during which Mr. Fred. J. Green has filled the post of sub-secretary to the Bath Eastern Dispensary. A permanent official in fact as well as in name, he has served as assistant to no fewer than eleven honorary secretaries in succession. This remarkable tenure of office has been acknowledged by the dispensary committee and placed on record in their latest report, and Mr. Green has been elected a life governor of the institution. The present honorary secretary, Mr. F. Ernest Shum, has held the post for several years, and the dispensary itself is an old one, having been established so long ago as 1832. Mr. Green, then, has not held, as is sometimes the case, a post on the permanent administrative staff from the beginning of the institution, but his period of office is none the less remarkable as an instance of the grateful service and continuity of interest which the voluntary system evokes.

### A HOSPITAL IN THE ANTARCTIC.

Dr. A. H. Macklin, who resigned his position as senior house surgeon at the Blackburn and East Lancashire Royal Infirmary in order to join Sir Ernest Shackleton's Trans-Antarctic Expeditionary Party, has written some entertaining descriptive letters to his father Dr. Macklin, of Whalley (Lancs.). In one of his letters, forwarded from South Georgia, a British Dependency in the South Atlantic, some hundreds of miles south-east of the Falkland Islands, Dr. Macklin mentions that he has been housed for a month in the isolation hospital, which was placed at the disposal of the party by the manager of the whaling station. As to hospital life on this fringe of civilisation, it appears that on Dr. Macklin's visit the hospital was unoccupied by patients—but he will, no doubt, have learnt something of the way in which sickness is coped with in that region. The hospital itself is built entirely of wood, covered with corrugated iron, and as the ground is continually damp from the melting snow, it is raised about two feet on iron posts. There are four rooms, all opening off a passage running from the front door to the back door, and each room has two windows and a stove. Outside there is a small verandah, and the hospital is fitted with electric light. The sea is ten yards away, and even nearer than that is a swiftly-flowing clear stream whose waters run straight down from the snows. The beds in the hospital are the simple, aseptic, easily detachable beds of a fever hospital, and quite comfortable to sleep on. "This," says Dr. Macklin, "is absolutely our last peep at civilisation, and it is a pretty crude civilisation here. We were very lucky to get this hospital to live in, with its luxury of spring beds. I do not know how I shall like my bunk when I go back to the ship." It is a very long stride from the wards of a Lancashire hospital to the open of the Antarctic, and the few medical men who have successfully passed through experiences such as those which Dr. Macklin is now doubtless enjoying, have already recorded observations of the utmost interest.

### THE SATURDAY FUND IN WAR TIME.

The first meeting for the present year of the reconstituted Board of Delegates of the Metropolitan Hospital Saturday Fund was held on the 17th inst., when it was stated by the secretary that the Board, so far, consisted of 219 members—viz., eighty-nine representing contributing firms; seventy-nine local committees, and fifty-seven representing participating institutions. Sir Thomas Vezey Strong was re-elected chairman of the Association; Mr. J. Neal was chosen chairman of the Council, and the Hon. H. L. W. Lawson, M.P., was reappointed hon. treasurer, with Mr. B. Marwood as hon. sub-treasurer. Other appointments were: Mr. F. Curtis, hon. standing counsel; Mr. H. Owen, hon. solicitor; and Messrs. J. H. Dilley, J. Summers, D. Murdock, A. H. Rundell, and Simpson, hon. secretaries. "Hospital Saturday" was fixed for October 9. The receipts to the 17th inst.—£1,541—were £61 in advance of last year at the same date. During the past fortnight the number of benefits issued have exceeded one thousand a week.

### A PIONEER MEDICAL SUPERINTENDENT.

A remarkable period of varied initiative and public service comes to an end now that Dr. R. A. Birdwood, M.A., M.D. Cantab., M.R.C.S., superintendent of the Park Fever Hospital, Lewisham, under the control of the Metropolitan Asylums Board, has resigned his position. He entered the service of the Board as an assistant medical officer in January 1878, leaving in May of the same year, but rejoined the service in June 1884, when he was appointed medical superintendent of the hospital ships, a post which he held until August 1892. At the time of his appointment in 1884, when convalescent smallpox patients only were removed out of London, he recommended and urged the removal of all smallpox patients direct from their homes to the ships. He pointed out that the adoption of this advice would inevitably result in a diminution or prevention of the incidence of smallpox. Expert advice in an opposite direction was given, as it was stated that patients would die on the voyage to the ships, that smallpox would not be prevented, and that it was impracticable. At length, in 1886, however, the Managers adopted Dr. Birdwood's recommendation, and of the success of that policy there can be no question. Dr. Birdwood also carried out certain alterations in the dietary of the smallpox patients which resulted in a reduction alike of mortality and expenditure. This practice he subsequently extended to other infectious patients, and it was generally adopted. In August 1892 Dr. Birdwood was entrusted with great responsibility in the planning, opening, and furnishing of the North-Eastern Fever Hospital, which was erected in the course of a few weeks at a time of great pressure. He has also acted similarly in connection with the opening and equipment of the Park Fever Hospital, and carried out its conversion from the hospitals to the children's service. Recently he has carried out its re-conversion for infectious diseases.

### ST. MARK'S AS A CANCER HOSPITAL.

It seems that some exception has been taken to the inclusion of the term " cancer " in the title of the hospital known officially as " St. Mark's Hospital for Cancer, Fistula, and Other Diseases of the Rectum," and the point was referred to at the recent annual meeting by the chairman, Sir Richard Biddulph Martin. We sympathise with the claim of the hospital to use this term as an accurate description of the hospital's activities. On what grounds can the phrase be challenged? Can anyone deny that the staff is competent to deal with cases of cancer of the rectum, or that as a matter of fact it does deal with such cases? There are, of course, hospitals which are devoted to the treatment of cancer, and are known under this name. But this does not mean that such institutions have any exclusive right to the title, or to the class of work which the title indicates; and any claim in this direction ought to be strenuously resisted. It seems to be a part of human nature that when a privilege is granted there arises a desire to convert it into a monopoly. Special hospitals have their justification, but must not be allowed to suggest that they alone are competent to undertake the work expressed in their official titles. By such titles they voluntarily limit themselves to particular departments, but this does not imply that they may restrict the activities of other and more comprehensive institutions.

### SHOULD THE POOR LAW SUBSIDISE SANATORIA?

The Wandsworth Board of Guardians have decided to call a conference of representatives of Metropolitan Poor-Law Authorities to consider the method of opposing the precepts of the Metropolitan Asylums Board so far as they include the payments for acquiring land, the construction of buildings and their equipment and maintenance for sanatoria, and of the treatment of patients out of Poor-Law funds. It is maintained that under Clause 64 of the National Insurance Act these payments should be made out of Government or County funds, and that the Poor Law should not in any way be called upon to subsidise the sanatoria. Mr. D. Grundy, a member of the Wandsworth Board of Guardians, who was responsible for a resolution being sent to the other Metropolitan Boards of Guardians condemning the proposed action of the Metropolitan Asylums Board, which was adopted by two-thirds of the authorities, pointed out at a meeting of his Board that the whole position was due to the London County Council in failing to fulfil its obligations, and in handing them over to the Metropolitan Asylums Board to carry out. The principle involved was that by the Metropolitan Asylums Board's precept, whether sanctioned or not, they were entirely taking out of the control of the ratepayers the supervision of the spending of the money subscribed by them for sanatoria treatment in the County of London. The conference is to meet within the next six weeks, when the question will be discussed as to whether it will be advisable to obtain the decision of the Law Courts on the legality of the Metropolitan Asylums Board in spending money out of the Poor-rates for the erection of sanatoria.

### THE EPSOM HOSPITAL AFFAIR.

As a result of a conference between the trustees of the Grand Stand Association, Epsom, and the committee of the military hospital which has been occupying the annexe, arrangements have been made to enable the patients to remain, notwithstanding the original agreement, recorded last week, that they should vacate the annexe during the spring and summer race meetings. A compromise has been arrived at, but the difficulty should have been provided for before the annexe was ever accepted for hospital purposes.

### THE "BLOCKADE" AND THE DRUG MARKET

The market continues active, and the general tendency of prices is still in an upward direction. In fact, during the past week this upward movement has been rather more pronounced; this, however, cannot be attributed to the " blockade," but is merely due to the fact that some of the drugs in most common demand are getting scarcer and scarcer owing to the gradual depletion of limited supplies. As a matter of fact, the threatened " blockade " has had little, if any, effect on the market. Phenacetin, antipyrine, and salicylates are among the drugs which continue to advance in price. Cod-liver oil is dearer, notwithstanding that the results of the Norwegian fishing have, up to the present, been more favourable than had been anticipated at the beginning of the season; the advance is mainly due to the large demand from Germany for oil and the risks to shipping from the producing districts; the probability is that prices will advance still further. Quotations for calomel and corrosive sublimate have now been raised in consequence of the further advance in the value of quicksilver. Citric acid, tartaric acid, and essence of lemon have a slightly downward tendency in price. Boric acid is dearer, and the prices of sodium benzoate and benzoic acid have advanced slightly.

